# Supplementary material for: Analytical framework to evaluate and optimize the use of imperfect diagnostics to inform outbreak response: Application to the 2017 plague epidemic in Madagascar
Source: PLoS Biol. 2022 Aug 15;20(8):e3001736. doi: 10.1371/journal.pbio.3001736 (PMC9410560; doi:10.1371/journal.pbio.3001736)
Supplement: S1 Text — Fig A. Molecular biology (MB) algorithm. Fig B. Model fit as a function of covariance between qPCRpla and qPCRcaf1 sensitivities for pneumonic forms (A) and bubonic forms (B). Fig C. Sensitivity of parameter estimates to different levels of correlation between the sensitivity of qPCRpla and qPCRcaf1. Fig D. Performance of case classification system assuming sample quality of 75%. Fig E. RDT vs. MB concordance over time. Fig F. Model fit as a function of the timing of changed RDT performance for pneumonic (PP) (A) and bubonic plague (BP) (B). Fig G. ROC plots presenting for a range of possible classification criteria for pneumonic (PP) (A, C) and bubonic plague (BP) (B, D, E) before (A, B) and after week 41 (C, D) and during the 2018 endemic season (E). Fig H. Distribution of positive predictive values (PPVs) by test result and clinical form. Fig I. Traceplots for MCMC of default model for pneumonic forms. Fig J. Traceplots for MCMC of default model for bubonic forms. Table A. Model estimates of the performance of RDT, culture, MB, and of tests that would be based on single diagnostic outcomes. Table B. Model estimates of test performance of RDT, culture, MB, and of tests that would be based on single diagnostic outcomes. In addition to the default analysis presented in Table A in S1 Text, here, the initial cPCR was included in the analysis. Results of this test were removed from the final analysis because performances of that test were too low. The results of the initial cPCR were not considered in the case classification. Table C. Model estimates of the performance of RDT, culture, MB, and of tests that would be based on single diagnostic outcomes, in a scenario change in RDT performance at week 41 of the outbreak. Table D. Model estimates of the performance of RDT, culture, MB, and of tests that would be based on single diagnostic outcomes, with a noninformative uniform prior on the prevalence of infection among notified cases. (DOCX) [file pbio.3001736.s001.docx]

**S1 Text**

**Figures**


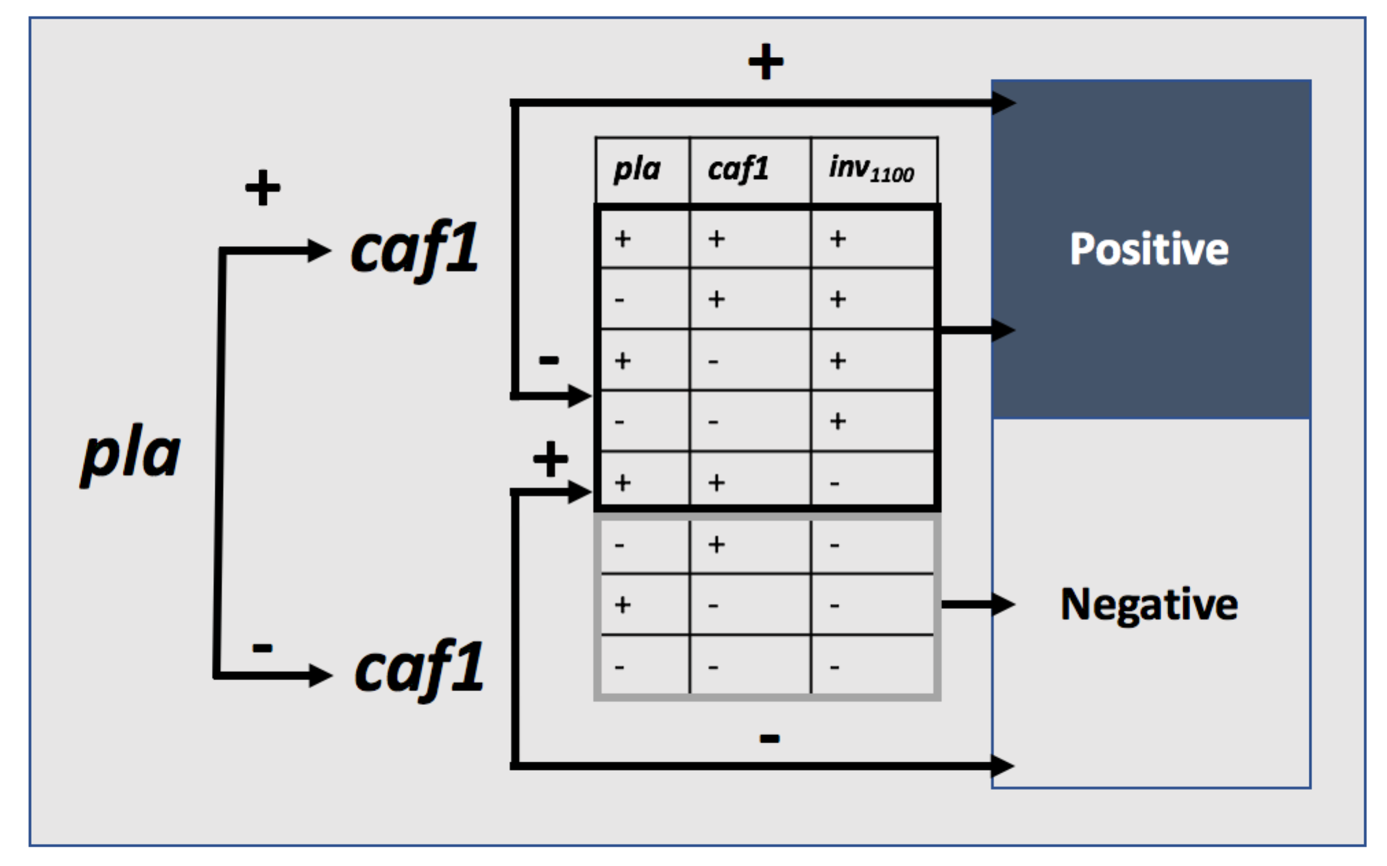


Fig A. Molecular Biology (MB) algorithm. All samples were tested for the presence of *pla* and *caf1* genes using qPCR. In case of discordance between two genes, an additional conventional PCR (box) targeting genes *pla, caf1,* and *inv_1100_* was performed. Samples were then considered positive upon positive results for *inv_1100_* and/or positive results for both *pla* and *caf1*.


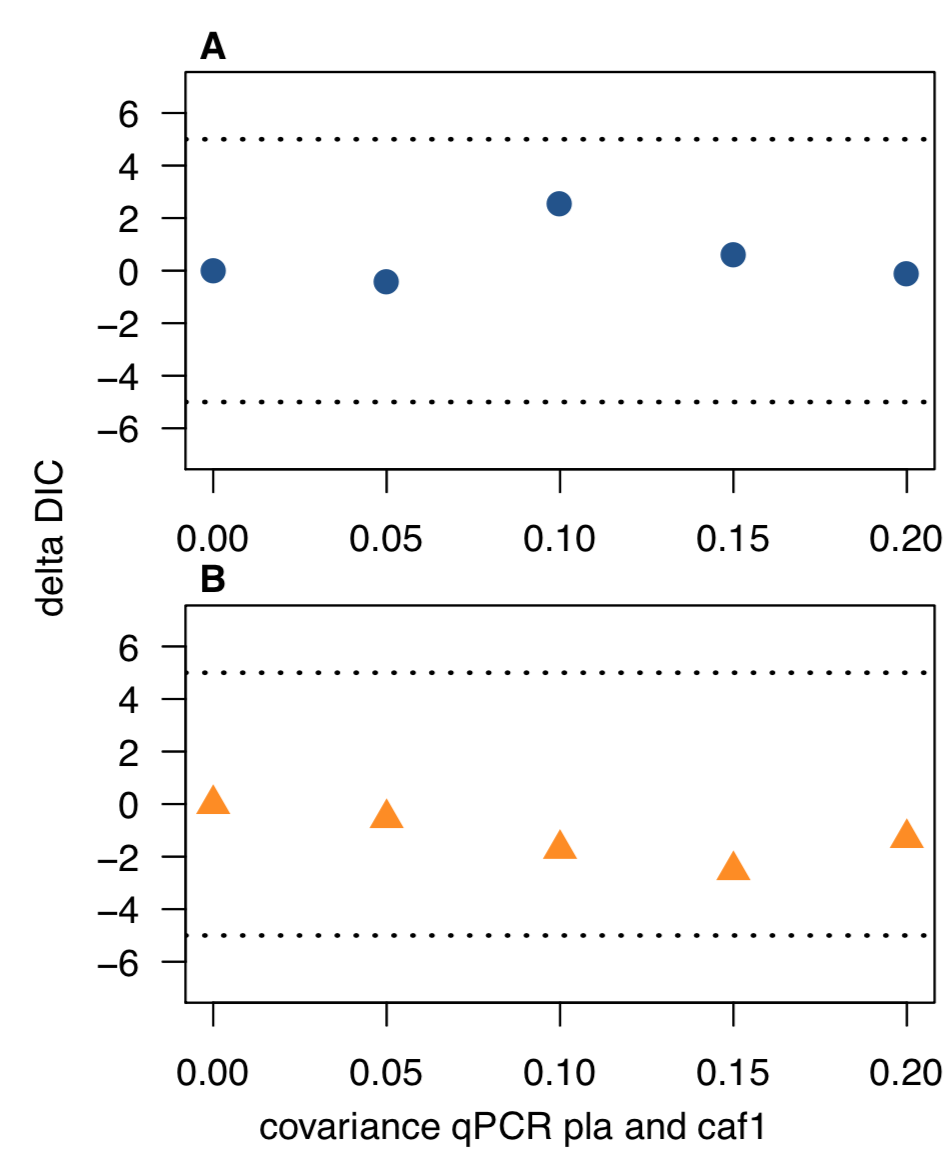


Fig B. Model fit as a function of covariance between qPCR*_pla_* and qPCR_c_*_af1_* sensitivities for pneumonic forms (A) and bubonic forms (B). DIC is the deviance information criterium, with lower values indicating a better model fit. Delta DIC denotes the difference between the baseline model (no covariance) and the models with increasing levels of covariance. Differences larger than 5 DIC (dotted line) are generally considered to depict significant improvement in model fit [1] . The underlying data and code to reproduce this figure are available on Open Science Framework (<https://osf.io/nbc4t/>).


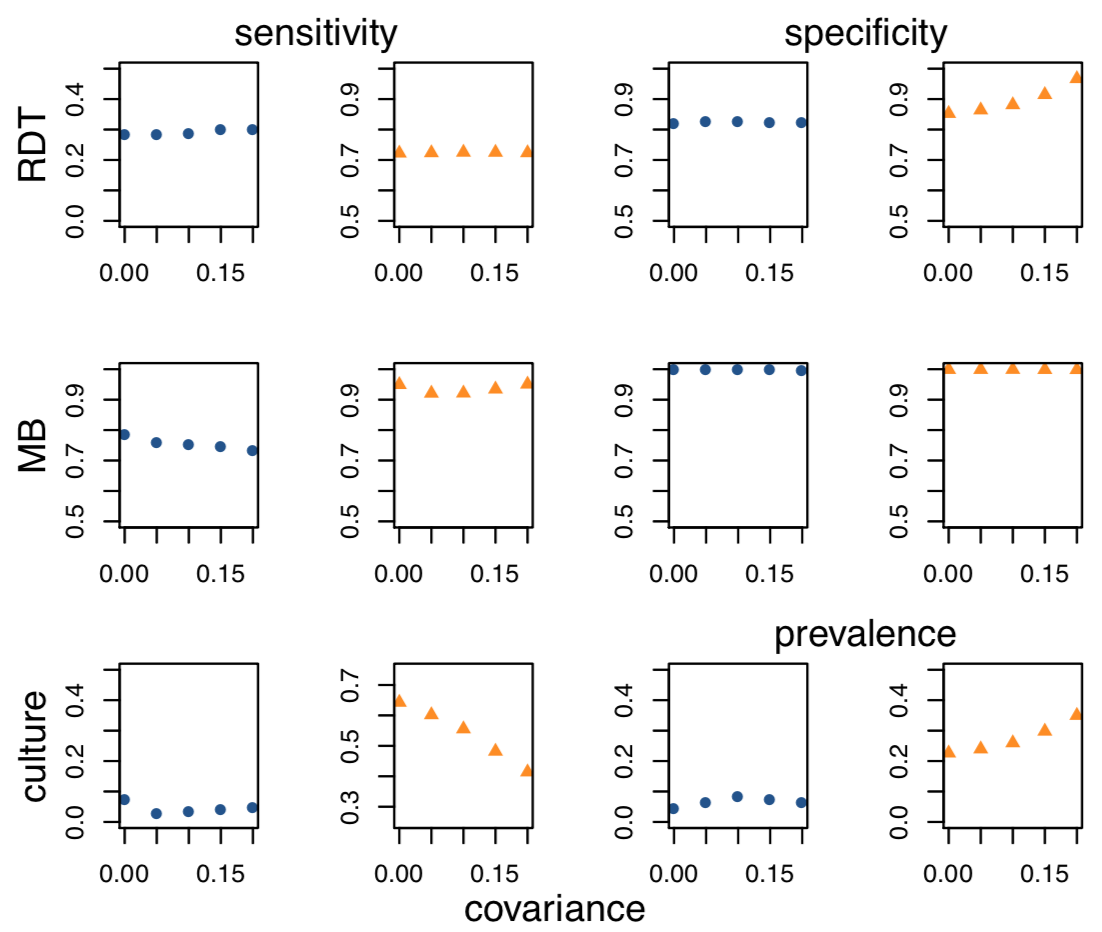


**Fig C. Sensitivity of parameter estimates to different levels of correlation between the sensitivity of qPCR_pla_ and qPCR_caf1_** for pneumonic forms (blue circles) and bubonic forms (orange triangles). The underlying data and code to reproduce this figure are available on Open Science Framework (<https://osf.io/nbc4t/>).


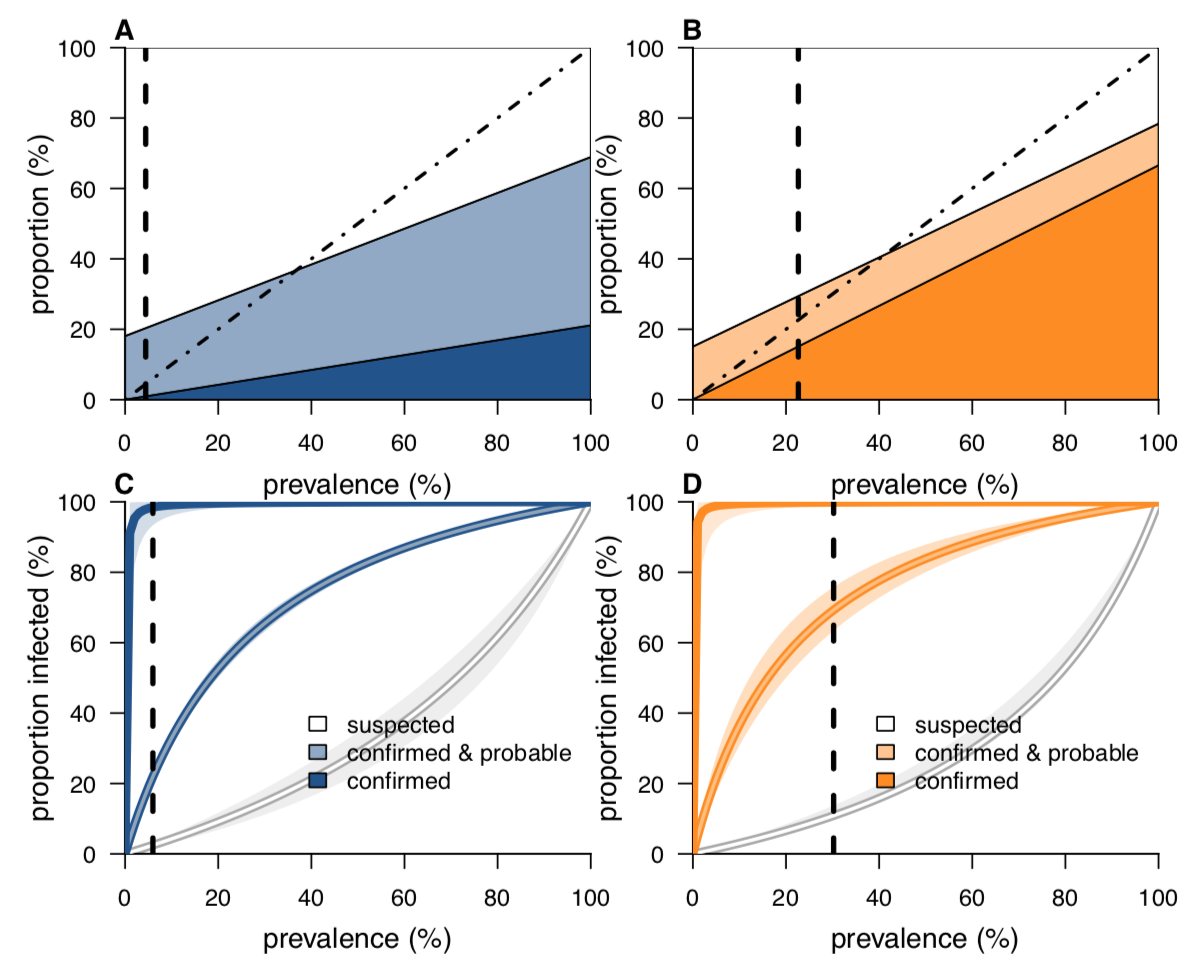


Fig D. Performance of the case classification system assuming a sample quality of 75%. (A,B) Expected proportion of notified cases classified as confirmed (dark blue or orange), probable (light blue or orange), and suspected (white), as a function of prevalence of infection for PP (A) and BP (B). The dashed vertical line indicates the prevalence among notified cases estimated during the 2017 Madagascar outbreak. The dashed diagonal line corresponds to perfect classification (C,D) Expected proportion of *Y. pestis* infections among cases in the category confirmed, confirmed or probable, and suspected as a function of prevalence of infection for PP (C) and BP (D). The underlying data and code to reproduce this figure are available on Open Science Framework (<https://osf.io/nbc4t/>).

**
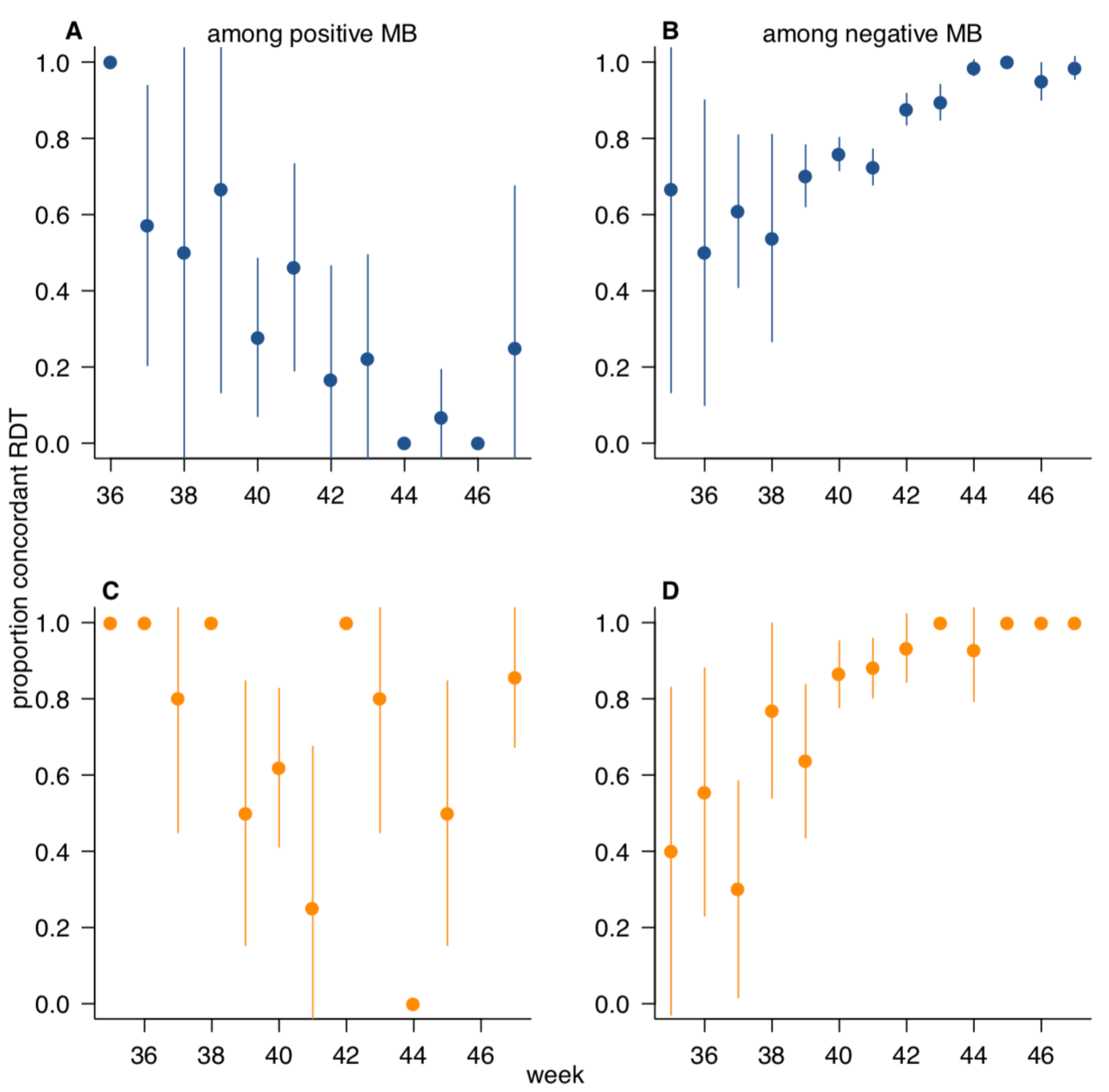
** **Fig E. RDT vs MB concordance over time.** Observed number of samples among positive (A,C) and negative molecular biology (MB) (B,D) with concordant results for the rapid diagnostic test (RDT). Pneumonic samples (blue: A,B) and bubonic samples (orange: C,D). The underlying data and code to reproduce this figure are available on Open Science Framework (<https://osf.io/nbc4t/>).


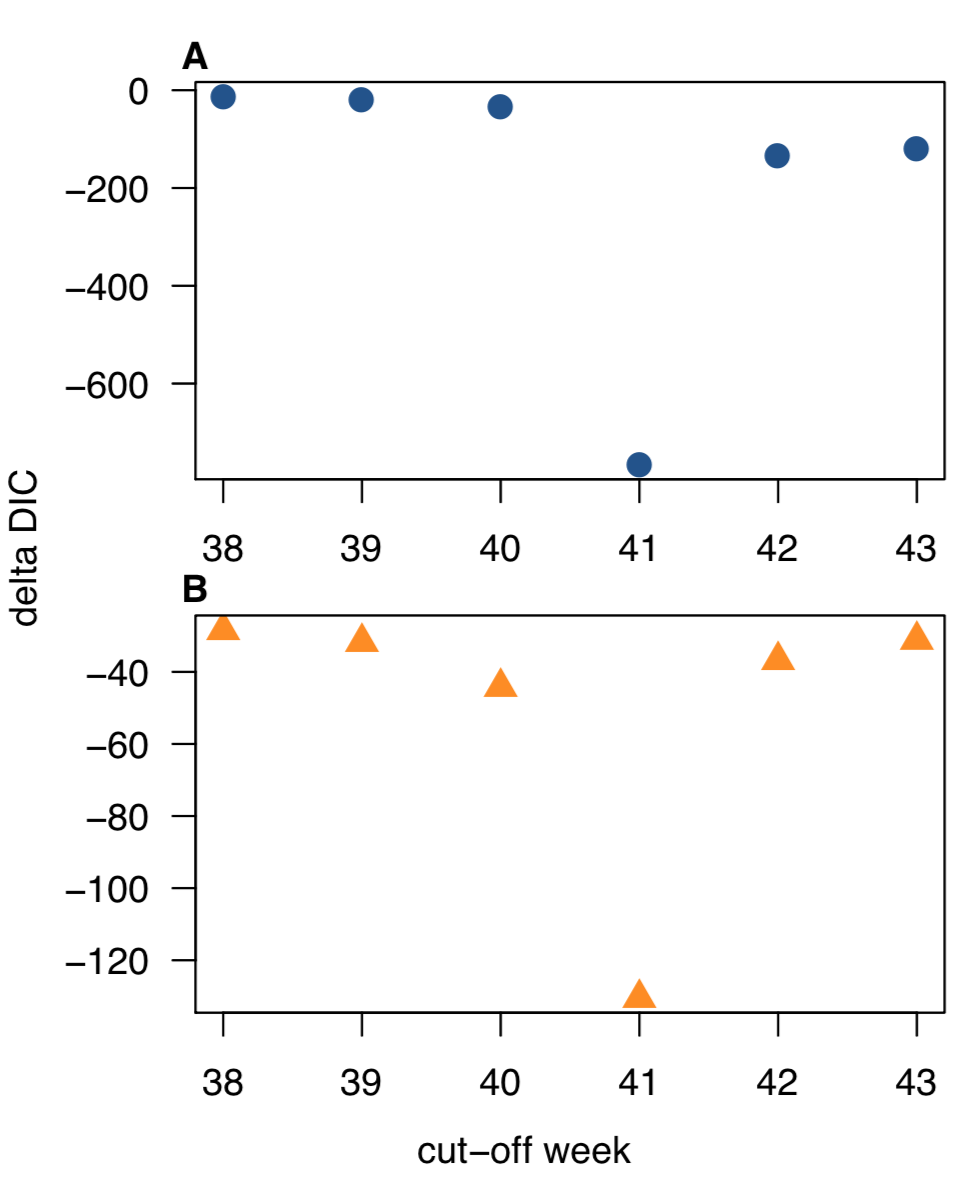


Fig F. Model fit as a function of the timing of changed RDT performance for pneumonic (PP) (A) and bubonic plague (BP) (B). DIC is the deviance information criterium, with lower values indicating a better model fit. Delta DIC denotes the difference between the baseline model (no change in RDT performance) and the extended models (step-change in RDT performance) with different cut-off times that this change may have occurred. As elsewhere in the manuscript, the timing of samples is determined by the date of symptom onset. The underlying data and code to reproduce this figure are available on Open Science Framework (<https://osf.io/nbc4t/>).


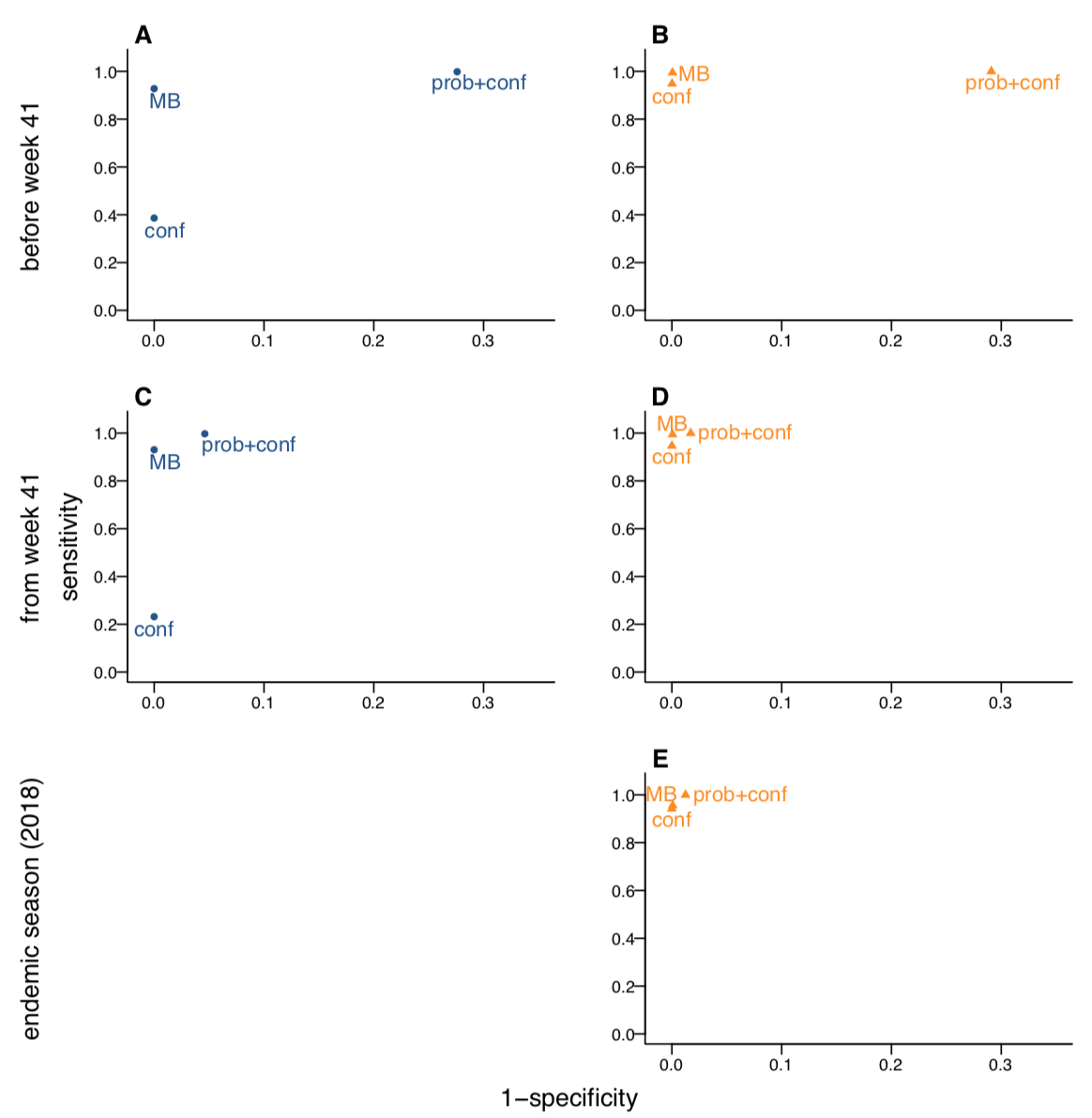


Fig G. ROC plots presenting for a range of possible classification criteria for pneumonic (PP) (A,C) and bubonic plague (BP) (B,D,E) before (A,B) and after week 41 (C,D) and during the 2018 endemic season (E). Classifications ≥1qpcr and 2qpcr represent results based on qPCR solely, i.e., in the absence of confirmatory cPCR, with ≥1qpcr denoting ‘at least one gene positive’ and 2qpcr ‘both genes positive’. The underlying data and code to reproduce this figure are available on Open Science Framework (<https://osf.io/nbc4t/>).


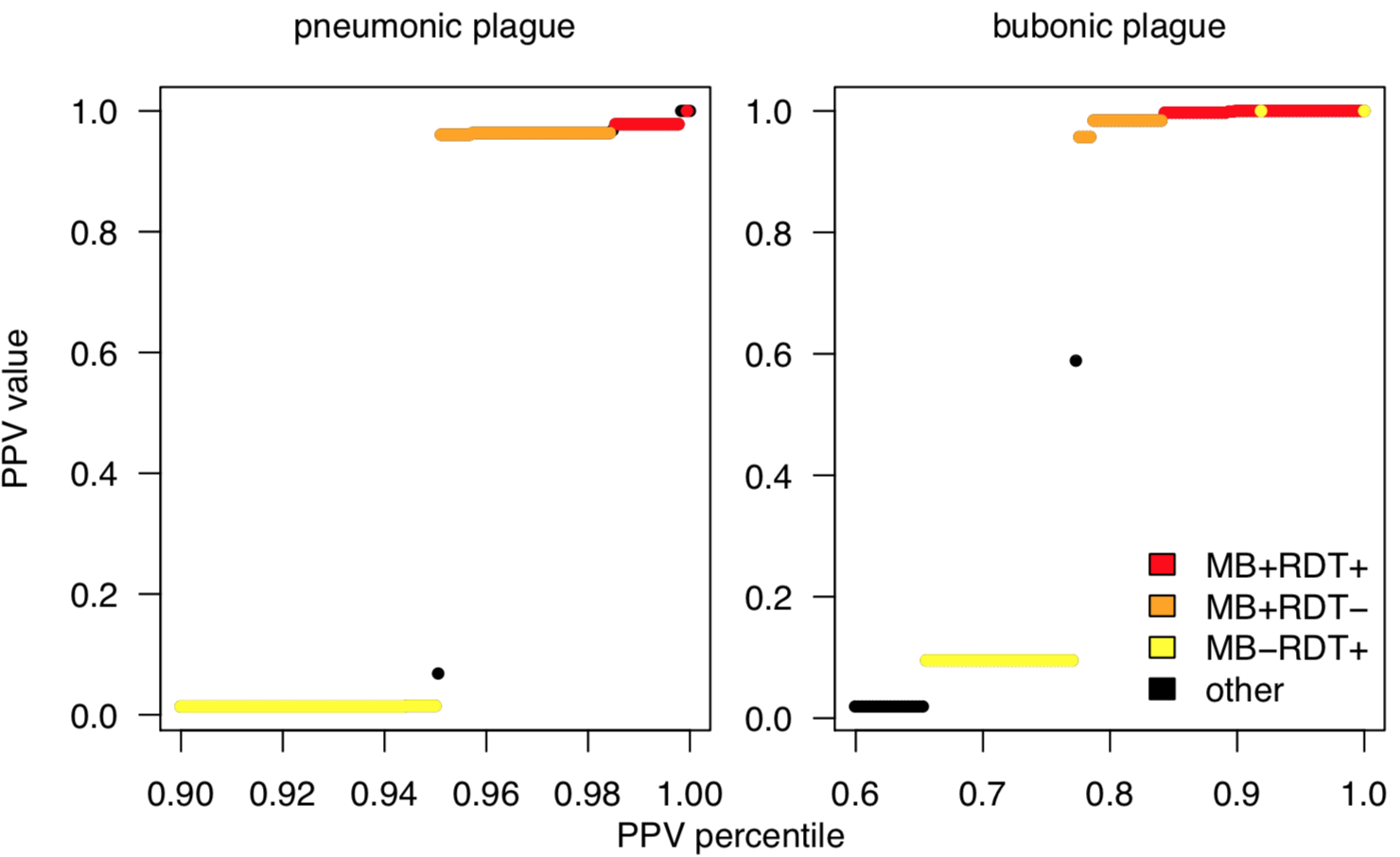


**Fig H. Distribution of positive predictive values (PPV) by test result and clinical form.** The probability of being infected with *Yersinia pestis* given a test result is calculated based on the estimated infection prevalence among notified cases and the estimated sensitivity and specificity of the diagnostic tests. Colors denote the results for molecular biology (MB) and rapid diagnostic tests (RDT). The underlying data and code to reproduce this figure are available on Open Science Framework (<https://osf.io/nbc4t/>).


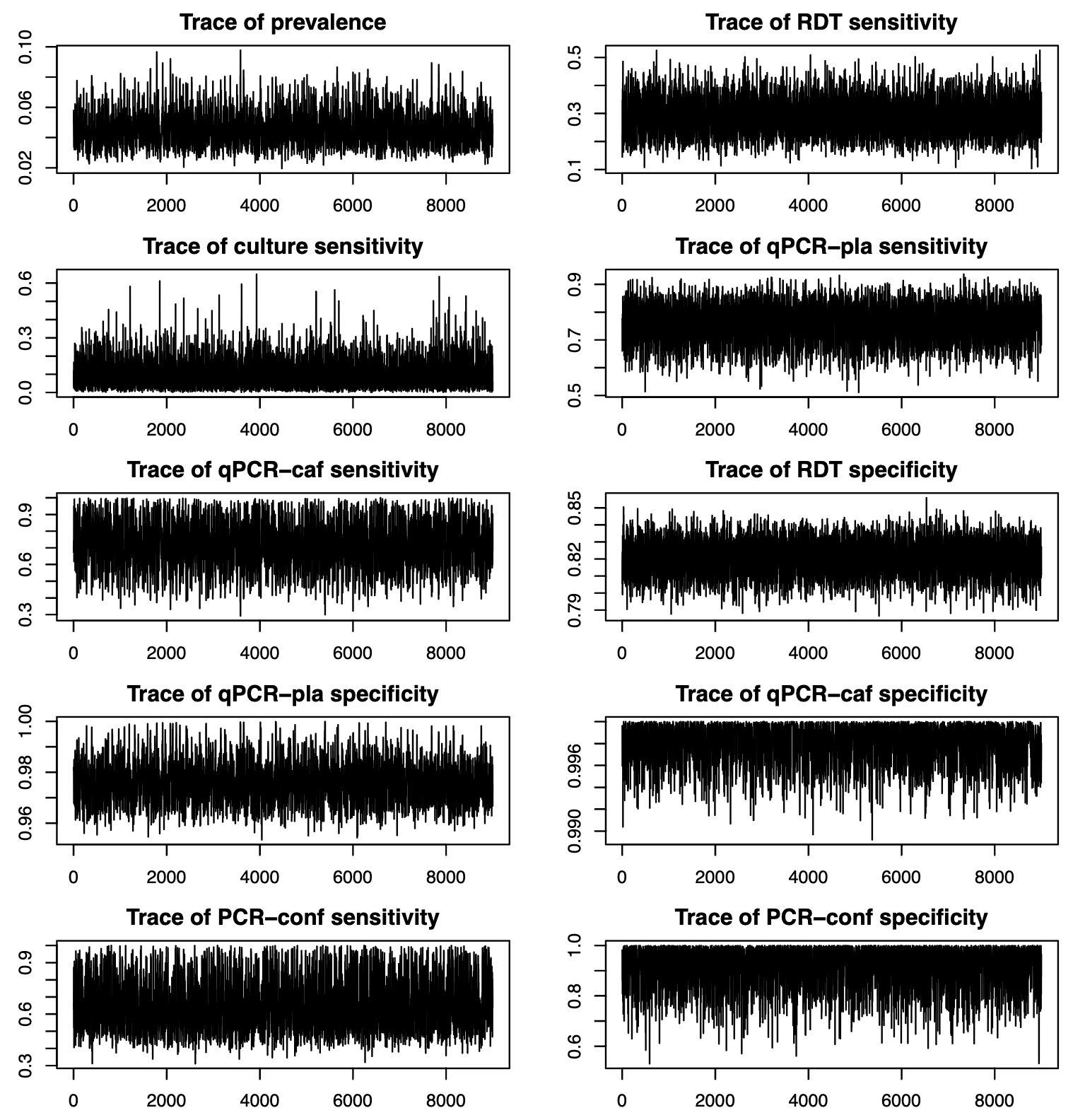


**Fig I. Traceplots for MCMC of default model for pneumonic forms with thinning equal to 10.** The underlying data and code to reproduce this figure are available on Open Science Framework (<https://osf.io/nbc4t/>).


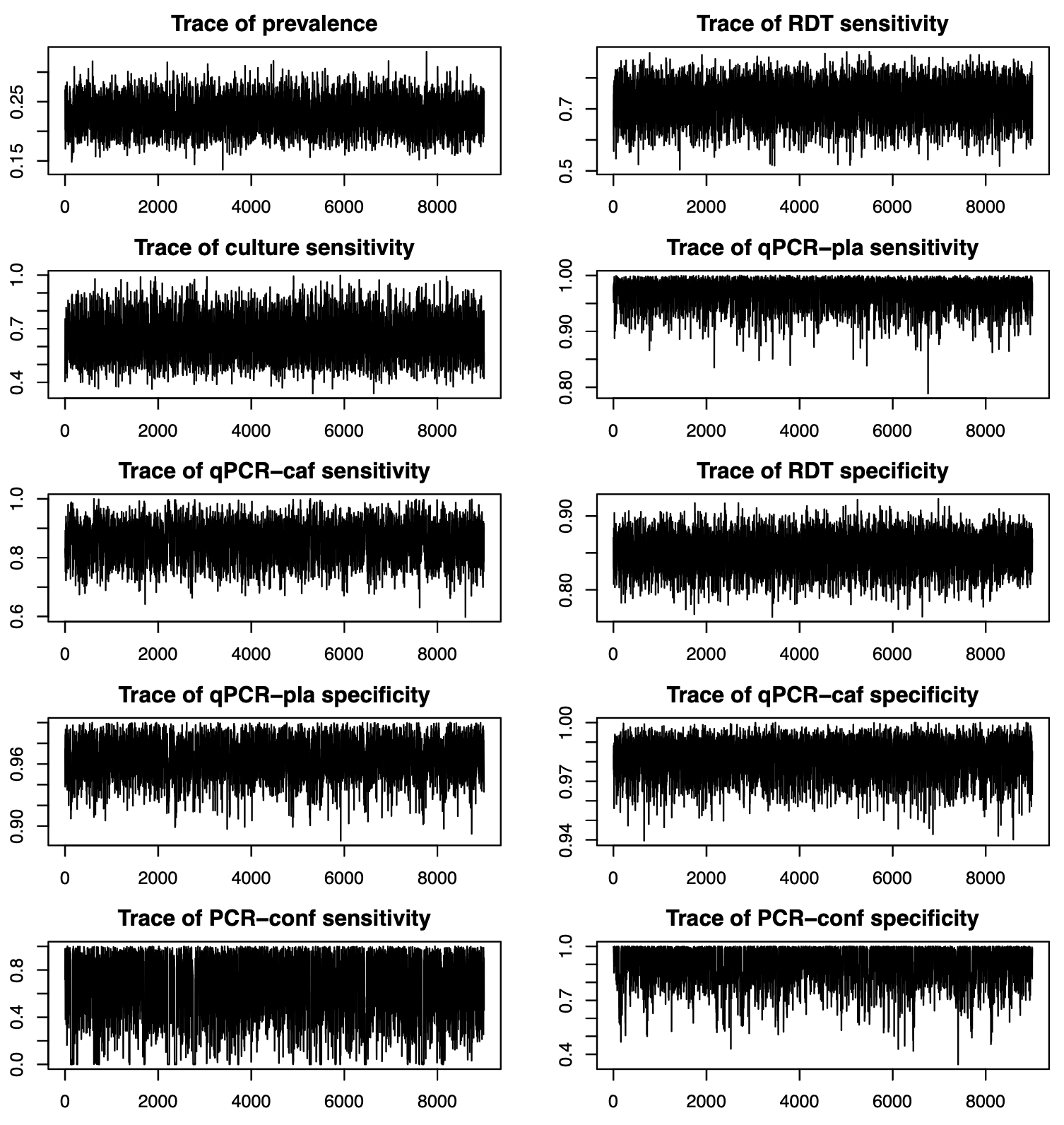


**Fig J. Traceplots for MCMC of default model for bubonic forms with thinning equal to 10.** The underlying data and code to reproduce this figure are available on Open Science Framework (<https://osf.io/nbc4t/>).

Table A. Model estimates of the performance of RDT, culture, MB and of tests that would be based on single diagnostic outcomes. The underlying data and code to reproduce this table are available on Open Science Framework (<https://osf.io/nbc4t/>).

| Diagnostic Outcome | Sens | | Spec | |
| --- | --- | --- | --- | --- |
|  | *Pneumonic* | *Bubonic* | *Pneumonic* | *Bubonic* |
| RDT | 28% ( 18- 41 ) | 72% ( 61- 83 ) | 82% ( 80- 84 ) | 85% ( 81- 89 ) |
| Culture | 7% ( 0- 23 ) | 64% ( 46- 85 ) | Fixed at 100% | Fixed at 100% |
| Molecular Biology^1^ | 80% (61-97) | 95% (86-100) | 100% (99-100) | 100% (98-100) |
| qPCR^2^ | 54% (32-76)^3^ | 84% (72-95) ^3^ | 100% (100-100) ^4^ | 100% (100-100) ^4^ |
| qPCR pla | 76% ( 63- 88 ) | 98% ( 93-100 ) | 98% ( 96- 99 ) | 97% ( 94-100 ) |
| qPCR caf1 | 71% ( 48- 98 ) | 86% ( 75- 97 ) | 100% ( 99-100 ) | 98% ( 97-100 ) |
| cPCR^5^ (confirmatory) | 63% ( 44- 95 ) | 69% ( 0- 97 ) | 95% ( 78-100 ) | 94% ( 72-100 ) |

^1^ Molecular biology algorithm based on qPCR on genes *pla* and *caf1* with, upon discordance, confirmatory cPCR on *pla*, c*af1*, *inv_1100_* genes ^2^ qPCR based on *pla* and *caf1* genes, ^3^positive result considered when both genes test positive, ^4^negative result considered when neither genes test positive, ^5^confirmatory cPCR based on *pla*, c*af1*, *inv_1100_* genes. Performed when qPCR result is inconclusive.

Table B. Model estimates of test performance of RDT, culture, MB, and of tests that would be based on single diagnostic outcomes. In addition to the default analysis presented in S1 Table in S1 Text, here the initial cPCR was included in the analysis. Results of this test were removed from the final analysis because performance of that test were too low. The results of the initial cPCR were not considered in the case classification. The underlying data and code to reproduce this table are available on Open Science Framework (<https://osf.io/nbc4t/>).

| Diagnostic Outcome | Sens | | Spec | |
| --- | --- | --- | --- | --- |
|  | *Pneumonic* | *Bubonic* | *Pneumonic* | *Bubonic* |
| RDT | 27% ( 17- 39 ) | 73% ( 63- 83 ) | 82% ( 80- 84 ) | 85% ( 81- 89 ) |
| cPCR pla (initial) | 86% ( 73- 97 ) | 95% ( 86-100 ) | 55% ( 52- 57 ) | 62% ( 55- 69 ) |
| Culture | 7% ( 0- 20 ) | 65% ( 47- 86 ) | Fixed at 100% | Fixed at 100% |
| Molecular Biology^1^ | 78% (52-96) | 95% (87-100) | 100% (99-100) | 100% (97-100) |
| qPCR^2^ | 48% (30-77)^3^ | 86% (75-97) ^3^ | 100% (100-100) ^4^ | 100% (100-100) ^4^ |
| qPCR pla | 81% ( 69-100 ) | 98% ( 94-100 ) | 98% ( 97-100 ) | 96% ( 92- 99 ) |
| qPCR caf1 | 58% ( 39- 77 ) | 88% ( 78- 99 ) | 100% ( 99-100 ) | 98% ( 97-100 ) |
| cPCR^5^ (confirmatory) | 70% ( 0- 96 ) | 68% ( 0- 97 ) | 95% ( 50-100 ) | 93% ( 68-100 ) |

^1^ Molecular biology algorithm based on qPCR on genes *pla* and *caf1* with, upon discordance, confirmatory cPCR on *pla*, c*af1*, *inv_1100_* genes ^2^ qPCR based on *pla* and *caf1* genes, ^3^positive result considered when both genes test positive, ^4^negative result considered when neither genes test positive, ^5^confirmatory cPCR based on *pla*, c*af1*, *inv_1100_* genes. Performed when qPCR result is inconclusive.

Table C. Model estimates of the performance of RDT, culture, MB and of tests that would be based on single diagnostic outcomes, in a scenario with a change in RDT performance at week 41 of the outbreak. The underlying data and code to reproduce this table are available on Open Science Framework (<https://osf.io/nbc4t/>).

| Diagnostic Outcome | Sens | | Spec | |
| --- | --- | --- | --- | --- |
|  | *Pneumonic* | *Bubonic* | *Pneumonic* | *Bubonic* |
| RDT- before week 41 | 33% ( 15- 53 ) | 73% ( 59- 87 ) | 72% ( 69- 76 ) | 71% ( 63- 78 ) |
| RDT- from week 41 | 14% ( 3- 30 ) | 72% ( 55- 88 ) | 95% ( 93- 97 ) | 98% ( 95-100 ) |
| Culture | 12% ( 0- 38 ) | 81% ( 63- 99 ) | Fixed at 100% | Fixed at 100% |
| Molecular Biology^1^ | 93% (85-100) | 99% (99-100) | 100% (100-100) | 100% (100-100) |
| qPCR^2^ | 54% (32-77)^3^ | 85% (73-98) ^3^ | 100% (100-100) ^4^ | 100% (100-100) ^4^ |
| qPCR pla | 77% ( 64- 88 ) | 98% ( 93-100 ) | 98% ( 96- 99 ) | 96% ( 93-100 ) |
| qPCR caf1 | 70% ( 46- 97 ) | 87% ( 77-100 ) | 100% ( 99-100 ) | 98% ( 97-100 ) |
| cPCR^5^ (confirmatory) | 64% ( 44- 96 ) | 68% ( 0- 97 ) | 95% ( 78-100 ) | 92% ( 67-100 ) |

^1^ Molecular biology algorithm based on qPCR on genes *pla* and *caf1* with, upon discordance, confirmatory cPCR on *pla*, c*af1*, *inv_1100_* genes ^2^ qPCR based on *pla* and *caf1* genes, ^3^positive result considered when both genes test positive, ^4^negative result considered when neither genes test positive, ^5^confirmatory cPCR based on *pla*, c*af1*, *inv_1100_* genes. Performed when qPCR result is inconclusive.

Table D. Model estimates of the performance of RDT, culture, MB, and of tests that would be based on single diagnostic outcomes, with a non-informative uniform prior from 0 to 1 on the prevalence of infection among notified cases. The underlying data and code to reproduce this table are available on Open Science Framework (<https://osf.io/nbc4t/>).

| Diagnostic Outcome | Sens | | Spec | |
| --- | --- | --- | --- | --- |
|  | *Pneumonic* | *Bubonic* | *Pneumonic* | *Bubonic* |
| RDT | 28% ( 18- 40 ) | 73% ( 62- 83 ) | 82% ( 80- 84 ) | 86% ( 81- 90 ) |
| Culture | 7% ( 0- 23 ) | 66% ( 47- 87 ) | Fixed at 100% | Fixed at 100% |
| Molecular Biology^1^ | 81% (59-100) | 90% (72-100) | 100% (99-100) | 100% (99-100) |
| qPCR^2^ | 53% (32-75)^3^ | 84% (72-94) ^3^ | 100% (100-100) ^4^ | 100% (100-100) ^4^ |
| qPCR pla | 76% ( 64- 88 ) | 98% ( 93-100 ) | 98% ( 96- 99 ) | 97% ( 94-100 ) |
| qPCR caf1 | 70% ( 46- 95 ) | 86% ( 81- 90 ) | 100% ( 99-100 ) | 98% ( 97-100 ) |
| cPCR^5^ (confirmatory) | 63% ( 44- 96 ) | 71% ( 15- 100 ) | 95% ( 79-100 ) | 94% ( 73-100 ) |

^1^ Molecular biology algorithm based on qPCR on genes *pla* and *caf1* with, upon discordance, confirmatory cPCR on *pla*, c*af1*, *inv_1100_* genes ^2^ qPCR based on *pla* and *caf1* genes, ^3^positive result considered when both genes test positive, ^4^negative result considered when neither genes test positive, ^5^confirmatory cPCR based on *pla*, c*af1*, *inv_1100_* genes. Performed when qPCR result is inconclusive.

**References**

1. Burnham KP, Anderson DR. (2003) Model selection and multimodel inference: A practical information-theoretic approach. : Springer Science & Business Media.stylefix
