# Supplementary material for: Analytical framework to evaluate and optimize the use of imperfect diagnostics to inform outbreak response: Application to the 2017 plague epidemic in Madagascar
Source: PLoS Biol. 2022 Aug 15;20(8):e3001736. doi: 10.1371/journal.pbio.3001736 (PMC9410560; doi:10.1371/journal.pbio.3001736)
Supplement: Attachment — Submitted filename: OptimizingDiagnostics_ReviewComments_PLOSBIOLOGY.pdf [file pbio.3001736.s002.pdf]

Reviewer #1: This manuscript employs Bayesian latent class analysis to better estimate the test characteristics of diagnostic tests used during an outbreak of plague in Madagascar, as well as to estimate the true prevalence of plague among reported cases. This type of analysis is increasingly being used to better understand medical diagnostics, particularly in cases for which no clear gold standard exists. The authors' work is novel; while the methods used are well-described they have not previously been applied to plague diagnostics. The manuscript is well-written and clear. The methods are also well-described; specifically the authors clearly delineate the prior distributions used for each parameter as well as the type of covariance structure used to evaluate the assumption of conditional independence. While one might have chosen different methods to model conditional dependence (e.g. the random effect model of Qu et al.), the authors' choice is reasonable and appropriate for this purpose.

We thank the referee for their positive comments.

Minor comments:
1.1. The authors did not mention what diagnostics were used to assess MCMC convergence; this should be explicitly stated in the methods.

Convergence was assessed visually. We added text to the methods section to make this explicit and added the corresponding trace plots (Figs S9 and S10)

Lines 387-388 (in Manuscript file without track changes, here and throughout): *"Traces of the MCMC were plotted per parameter and convergence was assessed visually (Fig S9 and S10). "*

1.2. The title for Table S2 would benefit from explicitly stating that this table was based on a model in which the prior for specificity was a uniform beta distribution (this is suggested in the text)

The reference in the text was incorrect. We apologize for this mistake. Table S2 is to show the analyses done that included the initial PCR that was performed (referred to in lines 118-120). We have updated the caption to reflect this. This now reads:
"Table S1. Model estimates of test performance of RDT, culture, MB, and of tests that would be based on single diagnostic outcomes. In addition to the default analysis presented in Table S1, here the initial cPCR was included in the analysis. Results of this test were removed from the final analysis because the performance of that test was too low. The results of the initial cPCR were not considered in the case classification. "
We had initially planned to add a sensitivity analysis on the assumption of perfect culture specificity, but because of the strong biological support of this assumption, we decided to leave this out and focus our attention on other sensitivity analyses.

1.3. Several of the supplemental figures were not referenced/mentioned in the text. Most of these were fairly well-explained, but it was not clear what Figure S9 was demonstrating. It would be helpful to have some mention of the supplemental figures either in the main text or perhaps in expanded/more descriptive figure legends (starting on line 491).

Thank you for this comment. Some of the Supplementary figures were indeed not or incorrectly referred to. We have careful check all Figures, identified and corrected labelling mistakes and ensured all Supplementary Figures are now referred to in the main text. We have also expanded the legend of Figure S9 (now S8) to make it more descriptive.

1.4. The authors state that they will make their code available on a specified github site. It would have been helpful for the review to have included the code as a supplemental file.

We agree it is important that the code is being made available. All code will be available on Open Science Framework. The reviewers can consult code and data at:
https://osf.io/fr3zc/?view_only=09db131b86684485a6ad07216e8919dd

Reviewer #2: The authors set out to address a major challenge in epidemiology: uncovering the spread of a pathogen when only imperfect diagnostic tests are available. I found the study to be well designed and enjoyed reading the manuscript. The approach used in this study has potential to be of broad interest for epidemiologists studying wide range of infectious disease. However, I do have some concerns, in particular with: i) the scope of the results, ii) the clarity/comprehensiveness of the Methods, and iii) the clarity of some figures. Provided these concerns can be addressed, I expect the manuscript to be suitable for publication in PLoS Biology.

We thank the referee for their constructive and thorough comments that have helped improve the manuscript.

Specific concerns:

Scope of results:

2.1. The estimates of prevalence of both BP and PP among notified cases seem very similar to their respective fractions of notified cases that are confirmed (i.e. confirmed using the imperfect diagnostic tests). I feel the study's importance would be much more evident if the deviation between estimated prevalence and fraction confirmed was clearly demonstrated, and its consequences explored. The authors state that "We showed that around one tenth of notified cases were likely to be infected with Yersinia pestis", but to my eye this looks comparable to the fraction of confirmed cases in the data (Figure 1C)?

We agree with the referee that the deviation between estimated prevalence and fraction confirmed is an important result and its consequences need to be fully explored. This is done by precisely quantifying this deviation and its impact both i) in the specific 2017 outbreak; but also, ii) in an extensive simulation study, considering other outbreak situations in which the prevalence of infection among notified cases could get any value between 0% and 100% and considering separately the examples of BP and PP (Figure 3 (now 4 in MS), see underneath). This systematic approach ensures we build a detailed understanding of the deviation between estimated prevalence and the fraction confirmed, when it is most problematic and when it works well, and how it compares with other classifications (e.g. “confirmed or probable”).

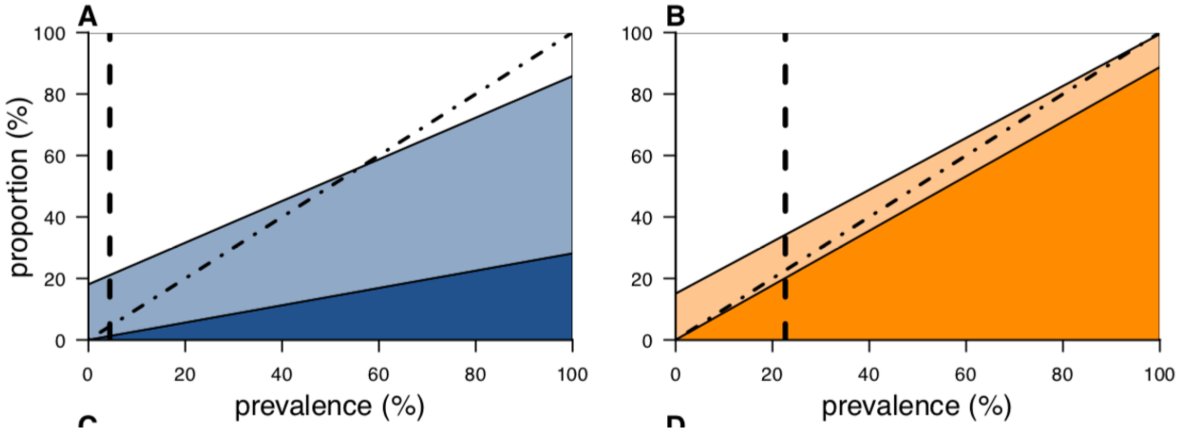

Figure 4. Performance of the case classification system. (A,B) Expected proportion of notified cases classified as confirmed (dark blue or orange), probable (light blue or orange), and suspected (white), as a function of prevalence of infection for PP (A) and BP (B). The dashed vertical line indicates the prevalence among notified cases estimated during the 2017 Madagascar outbreak. The dashed diagonal line corresponds to perfect classification

In the 2017 outbreak, the proportion confirmed was 2% among notified PP cases and 16% among notified BP cases, whereas the prevalence was estimated to be 4% (3-7%) in PP notified cases and 25% (18-28%) in BP notified cases. The reviewer is correct that the proportion of confirmed cases is the classification that gives the best indication of the overall prevalence, yet it underestimates the true burden of infection by 50% for PP and by 36% for BP.

We show in the simulation study presented in Figures 4 A and B above that this problem increases when the proportion of true infections among notified cases increases. In lines 138-146 (see below) and in Figure 4, we address the consequences of imperfect case classification and illustrate how the proportion of confirmed cases will increasingly underestimate the true burden of the outbreak as the prevalence among notified cases increases. For example, the relatively strict criteria for the confirmed cases would result in a three-fold underestimation of the true burden of infection if 50% of notified PP cases were truly infected (Figure 4A). As shown in the simulation study, the problem is particularly problematic for PP. For BP, the true burden consistently lies between the proportion confirmed and the proportion confirmed or probable (Figure 4B), showing that for BP, current case classification algorithms provide a good indication of the true prevalence of infection among notified cases.

This is further explained in (lines 150-158):
"Our analytical framework can be used to assess the performance of the case classification. For example, it can explain why the prevalence of Y. pestis among PP notified cases is estimated to be lower than the proportion of confirmed or probable cases (Fig 3A). In a scenario of low prevalence, the suboptimal specificity of RDT means that classification for PP based on confirmed or probable cases is characterized by a proportion of false positives (approx. 1-specificity) that is large relative to the prevalence. In contrast, a classification that solely relies on confirmed cases consistently underrepresents the prevalence due to low sensitivity of RDT and culture. For BP, the case classification performs well at any prevalence level, with the true prevalence always falling between the proportion of confirmed and confirmed/probable cases (Fig4B, Fig S4B)."

We further expanded the discussion on this important point (lines 268-271):
"While the proportion of confirmed cases gave the best indication of the true proportion of infections, a large underestimation of the true burden of infections is expected in scenarios with less

overreporting and a higher prevalence among notified cases.”.

2.2. The author's results suggest that both "suspected" and "probable cases" are extremely unreliable indicators for whether an individual is infected with plague (with suspected being far worse). Do you know why both are so bad? Also could you state the diagnostic criteria used to determine whether someone is suspected vs. probable? I'm not an expect on plague diagnosis, but I would imagine some of the symptoms that medical practitioners are using to inform their diagnosis (of bubonic plague in particular) are pretty unique, so I'm surprised they are doing so poorly.

All notified cases are clinically suspected (lines 296-297). However, it’s important to clarify that, although there is a clinical case definition as prescribed by the WHO, the clinical case definition did not need to be satisfied to be tested. This point was added to the Methods section (lines 313-314): “There was no formal clinical case definition that needed to be satisfied for patients to be tested.”
The importance of good clinical case definition was further added to the discussion. (lines 263-264) ”Apart from upholding test performances, this may also include robust clinical case definitions to prevent large overreporting.”
And is addressed in the results section (lines 171-174):
“For example, if the prevalence of PP was 20%, over half of confirmed or probable cases would be expected to be Y. pestis-infected. This proportion drops to as little as 22% (21-24%) for a prevalence of 5%. This shows that it is critical to avoid overreporting and ensure notified cases meet the clinical case definition.”
Case classification among notified cases is subsequently done based on test results (Figure 2 and lines 328-331 in the Methods section):
“As per WHO guidelines, cases were classified based on their diagnostic test results as confirmed if culture and/or both RDT and MB were positive, probable upon positive results for either MB or RDT, and suspected otherwise (Figure 2). Initial cPCR results were not considered for case classification. Culture is often regarded as a gold standard given its perfect specificity, yet lacks sensitivity.”

Symptoms for BP are indeed specific, but this is much less so for PP (lines 72). This, together with raised awareness about the disease and panic surrounding the outbreak, among others, may have led to a large volume of notified cases that were not infected with *Yersinia pestis*. If diagnostic tools have low specificity, this becomes particularly problematic when the prevalence among notified cases is low. This was the case for PP in particular. Therefore, the majority of those that classified as probable were not truly infected with *Y. pestis* (lines 253-255):
“In the case of the plague outbreak in Madagascar, limited RDT specificity contributed to the majority of probable pneumonic plague cases not to be infected with Yersinia pestis.”

We have added some additional text to clarify the characteristics of notifications and case classifications:
Lines 328 ‘based on their diagnostic test results’
We have added text to clarify that all notified cases are clinically suspected, and that the further classification is solely based on test results:
Lines 76: added ‘ clinically suspected’ when referring to notified plague cases.
Lines 99: added ‘based on their diagnostic outcomes (Figure 2)’, when describing the case classifications
Lines 110: added ‘clinically suspected’ when referring to notified cases in the definition of prevalence

The need to respect a clinical case definition is one of the important results emphasized by our study, with important implications for the management of future plague outbreaks.

2.3. The poor sensitivity of both RDT and culture suggest there would be a massive underestimation of PP cases if they were used as primary diagnostic methods. Could you explain why this underestimation is not the case (I'm trying to compare Figure 1A with Figure 4G taking into account the results in Figure 2).

For culture it is indeed true that the true prevalence is underestimated because of poor sensitivity. For RDT however, the combination of suboptimal specificity and low prevalence among notified cases results in a large volume of false positive cases. In figure 3C (now 4C), we illustrate that for a prevalence as low as 4%, the confirmed + probable class is expected to be dominated by cases that are not infected by *Yersinia pestis* (80% of probable + confirmed cases were not infected) (e.g. lines 250-255):

“We highlight the importance of accurate classification algorithms and show that, particularly for diseases with non-specific symptoms and high risks of misclassification (e.g. due raised awareness, or non-familiarity with the disease among public health responders), classification based on tests with poor specificity can result in vast overestimations of the outbreak extent. In the case of the plague outbreak in Madagascar, limited RDT specificity contributed to the majority of probable pneumonic plague cases not to be infected with Yersinia pestis.”

And (lines 152-158):

“In a scenario of low prevalence, the suboptimal specificity of RDT means that classification for PP based on confirmed or probable cases is characterized by a proportion of false positives (approx. 1-specificity) that is large relative to the prevalence. In contrast, a classification that solely relies on confirmed cases consistently underrepresents the prevalence due to low sensitivity of RDT and culture. For BP, the case classification performs well at any prevalence level, with the true prevalence always falling between the proportion of confirmed and confirmed/probable cases (Figure 4B, Fig S4B). ”

2.4. To what extent do the sensitivities and specificities shown in Figure 2 deviate from what was known before the study? If the tests come with stated sensitivities and specificities (which may, in light of this study, be inaccurate) it would helpful to state them here.

The RDT was produced *in-house* at Institut Pasteur de Madagascar. This test was first evaluated mainly on BP samples in (1). Up until the 2017 outbreak, little was known about the performance of RDT in PP samples as BP is the more common clinical form.

More recent estimates of RDT performance come from two literature reviews that present pooled estimates of RDT performance sensitivity and specificity of about 100% and 70% respectively when using culture as a reference standard and 72-95% and 87-93% when using PCR as a reference standard (2,3). However, as these estimates are based on reference standards with suboptimal sensitivity (particularly for culture), we expect that these past estimates overestimated sensitivity and underestimated specificity (as results that are assumed false positive may well be true positives that were not detected by culture).

We reanalyzed the results of *(1)* using latent class models and found that RDT estimates for bubonic samples were quite consistent with our estimates: sensitivity of 64% and specificity of 93% from the data of *(1)* while we estimate them at 72% and 85%, respectively, from our data. (lines 196-200):
“Estimates of RDT specificity for the second part of the outbreak are consistent with those obtained for the subsequent endemic BP season, during which the same batch was used (specificity: 99%, 96-

100%), and are quite consistent with estimates from earlier evaluations of this test (64% sensitivity and 93% specificity based on latent class analysis) (11). ”

No formal evaluation on culture or molecular biology have been published yet, to the best of our knowledge.

Likelihood function and data:

2.5. Estimating diagnostic test performance. I found the derivation and explanation of the per case likelihood function to be well reasoned and mostly clear, but a few changes would improve clarity.
Firstly, on lines 302-305 the diagnostic outcomes are indexed "1,...U" and then introduced explicitly "(i.e. RDT, qPCR (pla and caf1)". The relationship between the different test listed and the indexing (j = 1,…,U) took a bit of time to figure out. I suggest flipping the order, i.e. introducing the four tests performed irrespective of other outcomes and then explain how they are indexed. Also, if U = 4 then explicitly state this (or leave out the parameter U and replace it with 4 throughout). Finally, and this may be a question of personal preference, but when I see "y_ij" my mind thinks elements of a square matrix (e.g. correlation or distance matrices). Given the two indices label different things, I suggest changing one to a non-neighbouring letter (e.g. a or µ)

We have adjusted the text to make this easier to follow by i) introducing the different parts of the likelihood in lines 344-348 and ii) by introducing the four tests before explaining how they are indexed.
“Here, we calculate the contribution to the likelihood of the different diagnostic outcomes. Test specific sensitivities and specificities are then calculated from the characteristics of the diagnostic outcomes that make up a specific test (MB in particular). We first discuss the likelihood for those diagnostic outcomes that are performed irrespective of other diagnostic outcomes (RDT, qPCR), followed by those that are performed conditional on other diagnostic outcomes (culture and confirmatory cPCR).”

We recognize that the indexing of Y_ij may be confusing for some readers. We did however end up keeping this as is for consistency with other latent class papers.

2.6. Eq. 8 is the likelihood per case. I suggest adding a subscript to the left-hand side to indicate this (e.g.. L_i).

We have made the suggested addition

2.7. The main text and figures suggest that various different disaggregations of the data were performed (e.g. by spatial location or time of sampling). I found which data were used in which fit a bit challenging. It would be helpful to summarise/list these in a section of the methods, and explicitly state what was done in each scenario (e.g. something along the lines "the MCMC inference procedure was re-ran for each dataset individually").

Thank you. We added the following text to the ‘Methods Section’ for clarification (lines 437-448):
“Outbreak reconstruction
We derived the probability of Y. pestis infection for each notified case based on the positive predictive value associated with their results and assuming the medians of the estimated prevalence, sensitivity, and specificity (see eq 6.). The sum of all PPVs denotes the expected number of true infections among notified cases. We used this relationship to reconstruct the number of expected infections by subgroup. We divided the notified cases according to the following

categories: i) by period, distinguishing the initial phase (weeks 34-38), the outbreak phase (weeks 39-43), and the end phase (weeks 44-48), ii) by week, iii) by zone, distinguishing endemic zones (plague-endemic districts apart from greater Antananarivo), greater Antananarivo (urban community of Antananarivo and the three neighboring districts), and Toamasina district, and iv) by age group (below and above 5 years of age). Using these, we estimated the prevalence and exact binomial 95% confidence interval of infection among notified cases by subgroup. ”

MCMC:

2.8. The authors should explicitly list of all parameters estimated (perhaps as a table with the priors also in a column). At present the methods state "[w]e used uniform priors between 0 and 1 for most parameters", but I am unclear what those parameters are.

We clarified this by flipping the order that this is discussed and explicitly stating for which parameters uniform priors are applied. (lines 378-386):
“We utilized a weakly informative Beta-distributed prior for prevalence (shape = 1, scale = 2) (i.e., chance of prevalence being below 50% is twice as high as above), based on estimates of prevalence from previous BP outbreaks (22). To confirm the robustness of the results to the choice of priors on prevalence, the MCMC was also performed with a uniform prior between 0 and 1 (Table S4). For specificities of tests associated with MB, Beta-distributed priors were used with means of 95% (shape = 12.7, scale = 0.67), based on verifications done in the IPM laboratories prior to implementation. The specificity of culture was fixed at 100%. To assess the sensitivity to this assumption, the MCMC was also performed with culture specificity as a free parameter (S4 Table). For all other parameters, we used uniform priors between 0 and 1 (i.e., for all sensitivities as well as the specificity of RDT).”

2.9. The authors should state the exact MCMC algorithm used (e.g. "the Metropolis-Hastings algorithm with No-U-Turn sampler"). Additionally the authors should state the implementation of the algorithm (e.g. "implemented in python package pymc3").

We clarified that we used a Metropolis-Hastings algorithm in line 377-378. The algorithm was implemented by ourselves. All code will be available on the open science framework directory. We have added references to the main packages we used at lines (449-451):
“Software All analyses have been performed in R. MCMC results have been processed using the coda and BayesianTools packages. ”

2.10. I'm a bit concerned by the use of a weakly-informative beta-distributed prior for prevalence, given it is "based on estimates of prevalence from previous BP outbreaks". One of the findings of this study is that estimates of confirmed BP prevalence can be wrong -- so what's stopping this previous study suffering from the same diagnostic issues as present in the 2017 plague season. Is it necessary to assume this prior distribution and how does it impact on the study findings?

We applied this weakly informative prior to reflect the prior knowledge on plague outbreaks. In particular, we recognize that because previous BP outbreaks can be considered as a best ‘case scenario’ due to better case recognition in plague endemic areas and during regular outbreak years (27% was confirmed, 44% confirmed and probable). The choice of the prior reflects this notion by putting somewhat more weight on the prevalences below 50% (lines 379):
“(i.e., chance of prevalence being below 50% is twice as high as above)”.
As suggested by the referee, we have added a sensitivity analysis to check whether our results are robust to the use of this weakly informative prior. Both test performance and prevalence among notified cases were robust to using a different prior (i.e., uniform) (prevalence PP: 4% (3-7) to 5%(3-

7), BP: 25% (18-28) to 22% (18-27)). These results were added to the supplementary materials and discussed in the methods section (lines 380-382):
“To confirm the robustness of the results to the choice of priors on prevalence, the MCMC was also performed with a uniform prior between 0 and 1 (Table S4).”
And in the results sections (lines 124-126):
“Estimates were robust for deviations from model assumptions including the inclusion of the initial cPCR (Table S2) and the use of a uniform prior on prevalence (Table S4).”

Figure 1

2.11. I was unclear exactly what the proportions in Figure 1D and E correspond to. Are they the number of samples with a positive test result divided by the number of notified cases? If so, eyeballing panel B suggests that ~40% is too high for bubonic plague and culture, even if all confirmed cases are confirmed via culture. Or is it the proportion of culture samples that were positive? I think the caption needs revising to make clear exactly what is being shown.

We thank the reviewer for this attentive remark. Indeed, results in figure 1C-E show the proportion positive among samples on which the respective test was performed. RDT and MB were done on all samples. For culture this was more restrictive (lines 323-325):
“All samples received before November 3 were re-tested using the molecular biology protocols (November – December 2017). In addition, culture was performed on all samples with positive RDT (20). PP samples from non-endemic regions received between September 11 and October 3 were cultured irrespective of RDT result.”
We have clarified the caption for figure 1C,D,E by adding (lines 87-91):
“(C,D,E): Proportion of notified cases classified as confirmed (conf) or probable (prob) (C), with a positive test result for RDT, culture, or MB (NB. only cases on whom the respective test was performed are considered in the denominator. No restrictions were put on the use of MB and RDT. Culture was only performed if RDT was positive, apart from PP samples from non-endemic regions. On those samples, culture was performed irrespective of RDT result)”

Furthermore, given the y-axis ranges aren't that different between panels C-E, I suggest making them all the same.

We agree and have made the suggested change.

2.12. Are the "confidence intervals" really confidence intervals? The approach here is Bayesian — how are the confidence intervals calculated?

Thank you for spotting this mistake. We have corrected this. It now reads ‘credible intervals’

2.13. I wonder if panel A and B might benefit from being plotted on a log or square root scale.

We agree that this would increase the readability of the ‘confirmed’ results. However, we believe that the overall message of these two panels (i.e., the evolution of the volume of notified cases over time) is best conveyed on a linear scale.

Figure 2

2.14. Figure 2 would be improved by labelling all y-axis.

We have made the suggested changes.

Figure 3

2.14. I think clarity would be improved by changing the caption to read prevalence of infection amongst notified cases (consistent with rest of paper).

We have made the suggested change.

2.15. I take it the dashed diagonal line in A and B corresponds to a perfect classifier (sensitivity = specificity = 1). It would be helpful to mention this in the caption.

We agree and have added this to the caption (lines 162).

2.16. I enjoyed the ROC curve plots and found them very informative. However, I was unsure what "conf" and "prob" meant in this context (is it the same as confirmed and probable above?) and how their sensitivity and specificity were calculated. Furthermore, why was only molecular biology plotted here? Would't it be informative to also show the other diagnostic methods?

We have added the explanation of conf and prob to the caption (lines 166). Thank you for pointing this out.
The reason that separate diagnostic methods were not included here, was because this panel is devoted to the use of classification methods rather than diagnostic tools. MB was included into this figure because it is considered as a classifier by itself, due to its good performance. This was added to the caption (lines 165-166):
"MB is considered here due to its potential for being considered as a classifier by itself."
The sensitivity and specificity of a classification algorithm are estimated by calculating the conditional probabilities (i.e., conditional on being infected or not) for each test result that results in a specific classification.

Figure 4

2.17. Are these prevalence estimates among notified cases or overall population prevalence estimates (including cases that were not notified)?

These are infections among notified cases. This has been added to the caption.

2.18. It would be helpful to compare each panel to the respective data from each grouping. E.g. total fraction of notified cases that are confirmed/suspected.

Thank you for this suggestion. We have adjusted this figure accordingly.

2.19. The multi-y-axis in panels G and H fooled me for a long time. I spent a while confused how estimated infection among notified could be larger than notified. I suggest the authors plot both time series on the same y scale, maybe using a log or square root scale. If the authors want to stick with having a twinned y axis then the right-hand scale also needs labelling to make it clearer. Furthermore, I really think it would be helpful to also show the data on confirmed and suspected cases in these panels — i.e. what is shown in Figure 1A and B.

The choice for a secondary axes was made to highlight the relative changes over time. We do however agree with the referee that the absolute difference between notifications and estimated

true infections is less clear when presented this way. We have adjusted this figure as proposed: i) we have added the case classifications to figures G and H, and ii) we now present observed and estimated figures on the same axes. To make sure the latter is still insightful, we now present figures G and H in 'portrait.

Reviewer #3: This is a post-hoc analysis of an outbreak of plague caused by the bacterium Yersinia pestis in Madagascar in 2017 to 2018. The goal is to more accurately determine the size, timing, and spatial extent of the outbreak. The issue is that testing criteria for properly diagnosing plague generally have low sensitivity, especially for detection based on culturing of bacteria and a rapid diagnostic test based (RDT) on detecting antigens to proteins of the F1 capsule. Tests based on molecular biology (MB) use qPCR to detect the pla and caf1 genes have higher sensitivity. The problem is that the true gold standard, culturing of bacteria, has especially low sensitivity for sputum samples for pneumonic plague, and only slightly higher sensitivity for samples aspirated from lymph nodes for bubonic plague. A final MB qPCR test for a third gene inv1100 is highly sensitive and makes final confirmation more secure.

The main conclusion is that the best estimates for prevalence of confirmed and probable cases among suspected cases was only 5% for pneumonic plague, and 25% for bubonic plague. Thus, the outbreaks were not as large as initially suspected and reported. Further, neither culturing nor RDT provided reliable information. Higher accuracy in the case of both tests could have affected public health responses to the outbreaks. qPCR tests, especially for all three gene fragments, provide the most reliable testing. Nonetheless, the problem is still whether the testing would have provided actionable decisions, based both on how quickly qPCR tests can be turned around, and because prophylactic antibiotic treatment during outbreaks likely both suppressed the outbreak and affected the qPCR test sensitivity.

We agree that this is a great challenge in outbreak control. In this specific case, the allocation of resources (for instance mobile test facilities) is informed by the volume of notified cases classified as probable or confirmed. Therefore, a reliable case classification system is needed to optimize the impact of these resources. Similarly, contact tracing efforts can help mitigate the outbreak, but need to be implemented in the right place to have the most impact. We have added text about this to the discussion (lines 286-288):
"Improved case classification is particularly important for the allocation of scarce resources, for instance by accurately targeting contact tracing efforts and optimizing the impact mobile test facilities."

The study nonetheless provides a good framework for analysis of outbreaks, if the sensitivity and specificity of the tests are known.

One aspect that is not discussed at all is whether effective testing could lead to more judicious use of antibiotics, and potentially help slow antibiotic resistance by the plague pathogen. Keeping off the antibiotic treadmill as one antibiotic after another loses its effectiveness is a major reason for more accurate and efficient testing.

Although treatment is not contingent on testing, the prophylactic use of antibiotics is likely to be related to the estimated community transmission and therefore closely related to case classification. We added text to highlight this (lines 272-275):

“In this outbreak, this was particularly relevant as widespread use of prophylactic treatment was observed in response to the large volume of notified cases. The real risks of resistance emergence associated with widespread use are another reason why accurate case classification is important. ”

The flow of the paper is difficult to follow. Putting the decision trees for deciding whether a specific case is probable or confirmed into the main text, rather than in supplementary materials, would be helpful.

We have made the proposed suggestion. The decision tree has now been moved to the main manuscript as figure 2.

References

1. Chanteau, Suzanne, et al. "Development and testing of a rapid diagnostic test for bubonic and pneumonic plague." *The Lancet* 361.9353 (2003): 211-216.

2. Jullien, Sophie, Harsha A. Dissanayake, and Marty Chaplin. "Rapid diagnostic tests for plague." *Cochrane Database of Systematic Reviews* 6 (2020).

3. Rajerison, Minoarisoa, et al. "Performance of plague rapid diagnostic test compared to bacteriology: a retrospective analysis of the data collected in Madagascar." *BMC infectious diseases* 20.1 (2020): 1-6.
